# Protocol for multi-scale light microscopy/electron microscopy neuronal imaging in mouse brain tissue

**DOI:** 10.1016/j.xpro.2022.101508

**Published:** 2022-08-18

**Authors:** Kenta Yamauchi, Takahiro Furuta, Shinichiro Okamoto, Megumu Takahashi, Masato Koike, Hiroyuki Hioki

**Affiliations:** 1 Department of Neuroanatomy, Juntendo University Graduate School of Medicine, Bunkyo-Ku, Tokyo 113-8421, Japan; 2 Department of Cell Biology and Neuroscience, Juntendo University Graduate School of Medicine, Bunkyo-Ku, Tokyo 113-8421, Japan; 3 Department of Oral Anatomy and Neurobiology, Graduate School of Dentistry, Osaka University, Suita, Osaka 565-0871, Japan; 4 Advanced Research Institute for Health Sciences, Juntendo University, Bunkyo-Ku, Tokyo 113-8421, Japan; 5 Department of Neuroscience, Graduate School of Medicine, Kyoto University, Kyoto, Tokyo 606-8501, Japan; 6 Department of Multi-Scale Brain Structure Imaging, Juntendo University Graduate School of Medicine, Bunkyo-Ku, Tokyo 113-8421, Japan; 7 Japan Society for the Promotion of Science (JSPS), Chiyoda-ku, Tokyo 102-0083, Japan

**Keywords:** Cell Biology, Microscopy, Neuroscience

## Abstract

An imaging technique across multiple spatial scales is required for extracting structural information on neurons with processes of meter scale length and specialized nanoscale structures. Here, we present a protocol combining multi-scale light microscopy (LM) with electron microscopy (EM) in mouse brain tissue. We describe tissue slice preparation and LM/EM dual labeling with EGFP-APEX2 fusion protein. We then detail Sca*l*eSF tissue clearing and successive LM/EM imaging. Our protocol allows for deciphering structural information across multiple spatial scales on neurons.

For complete details on the use and execution of this protocol, please refer to Furuta et al. (2022).

## Highlights

• Successive light microscopy/electron microscopy combined with a tissue clearing protocol
• Multi-scale neuronal imaging from macroscopic to nanoscopic neuronal structures
• Light microscopy/electron microscopy dual labeling resistant for tissue clearing
• Simultaneous interrogation of brain-wide circuit structure and synaptic connectivity

## Abstract (editor highlights)

Publisher’s note: Undertaking any experimental protocol requires adherence to local institutional guidelines for laboratory safety and ethics.

## Graphical abstract

**Figure:**
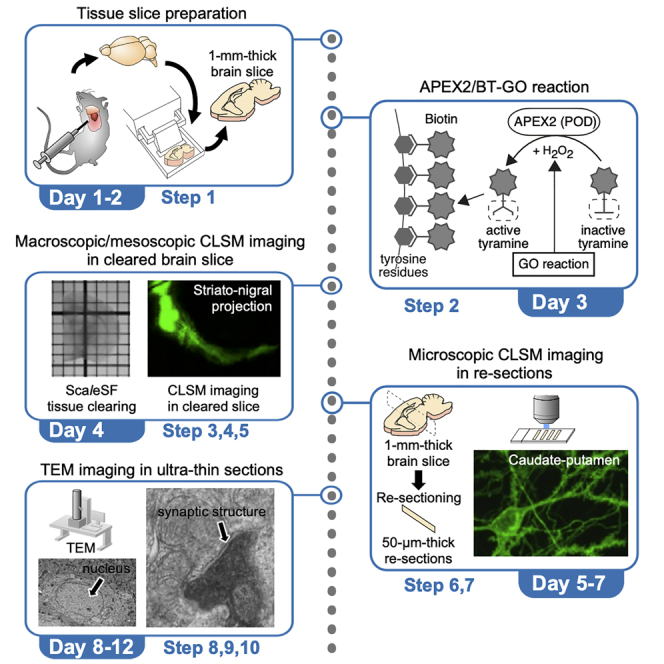

## Before you begin

The protocol below describes the specific steps for using mouse brain tissues. However, we have also used this protocol in brain tissues of common marmoset (*Callithrix jacchus*) (Furuta et al., 2022).

### Institutional permissions

All animal experiments in this protocol were approved by the Institutional Animal Care and Use Committees of Juntendo University (Approval No. 2020087, 2020088) and conducted in accordance with Fundamental Guidelines for Proper Conduct of Animal Experiments by the Science Council of Japan (2006). All experiments on live vertebrates or higher invertebrates must be performed in accordance with the Guideline for the Institutional Animal Care and Use Committee.

### Stereotaxic injection of an adeno-associated virus (AAV) vector

**Timing: ∼1 h**

For a detailed procedure, refer to Okamoto et al. (2021) and Takahashi et al. (2021).

1. Prepare AAV2/1-SynTetOff-EGFP-APEX2 vector solution (1.0 × 10^11^ infectious unit/mL).
2. Load the AAV solution into a glass micropipette.
3. Attach the micropipette to the holder of Picospritzer® III.
4. Anesthetize a C57BL/6J mouse (8–16 week old) by intraperitoneal (ip) injection of a mixture of Domitor® (0.3 mg/kg), Dormicum® (4 mg/kg) and Vetorphale® (5 mg/kg).
5. Mount the anesthetized animal onto a stereotaxic instrument.
6. Thin the skull with a dental drill above the injection site, and remove the remaining bone with fine forceps.
7. Insert the tip of the micropipette into the brain and eject the vector solution.
8. Close and sterilize the wound, and administrate gentamicin locally.
9. Recover the animal from anesthesia by ip injection of Antisedan® (1.5 mg/kg).
10. Maintain the animal for 4 weeks under regular health check.

### Preparation of a regent for tissue fixation

**Timing: ∼1 h**

11. 16% (w/v) paraformaldehyde (PFA) in distilled deionized water (ddH_2_O) (100 mL).
  a. Warm ∼80 mL ddH_2_O to 70°C–80°C.
  b. Add 16 g PFA to the warmed water.
  c. Dissolve PFA by adding 2 to 3 drops of 10 N sodium hydroxide (NaOH) solution.
  d. Allow the solution to cool to 20°C–25°C, and bring 100 mL with ddH_2_O.
  e. Filter the solution through filter paper.
  f. Dispense to 10 mL each.
  g. Store at −20°C. The solution can be stored at −20°C for 6 months.

**CRITICAL:** PFA is toxic and teratogenic. Avoid inhalation or contact with skin, eyes and mucous membrane. Handle it inside a fume hood with appropriate protective gear.

**CRITICAL:** NaOH is corrosive. Handle it with appropriate protective gear. Contact with skin, eyes and mucous membrane must be avoided.

### Preparation of an imaging chamber

**Timing: ∼3 h**

**Figure 1:**
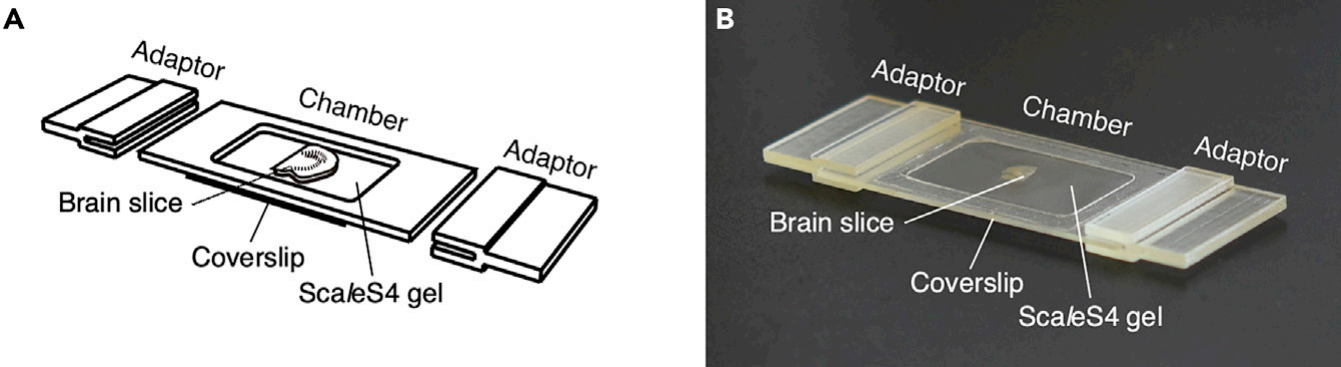
A customizable 3D-printed imaging chamber for optically cleared tissue slices (A and B) A schema drawing (A) and picture (B) of a customizable 3D-printed imaging chamber. The imaging chamber is composed of the chamber frame and bottom coverslip. Microscope stage adaptors are designed for mounting directly on a microscope stage. Sca*l*eSF-treated tissue slices are mounted onto the bottom coverslip and embedded in Sca*l*eS4 gel. The chamber frame and microscope stage adaptors are customizable. Reprinted and modified from Furuta et al. (2022) under the Creative Commons Attribution 4.0 International License (CC BY 4.0; https://creativecommons.org/licenses/by/4.0/).

A customizable three dimensional (3D)-printed imaging chamber is designed for reliable mounting of optically cleared brain slices (Figure 1) (Furuta et al., 2022). The chamber consists of the chamber frame and bottom coverslip. Microscope stage adaptors are also designed for direct mounting of the imaging chamber on a microscope stage. 3D computer-aided design (CAD) data for the chamber frame and microscope stage adaptors are provided in Furuta et al. (2022).

12. Prepare the chamber frame. The chamber frame is 3D-printed.

***Alternatives:*** Online 3D printing services (e.g., DMM.make [https://make.dmm.com]) provide a good alternative.

13. Secure the printed chamber frame to the bottom coverslip with a pressure-sensitive adhesive.

### Preparation of silicone-coated glass slides and coverslips

**Timing: 1 day**

14. Add 500 μL of Siliconized L-25 to 9.5 mL of chloroform, and mix well by stirring.
15. Spread the 5% Siliconized L-25 solution on glass slides and coverslips.
16. Let them dry.

**CRITICAL:** Chloroform is toxic and carcinogenic. Handle it inside a fume hood with appropriate protective gear. All necessary precautions should be taken when disposing.

### Preparation of rocket of resin

**Timing: 3 days**

17. Mix well 3.8 mL of Luveak-812, 2 mL of Luveak-DDSA and 2.4 mL of Luveak-MNA by slowly stirring for 5 min.
18. Add 0.12 mL of Luveak-DMP-30 to the mixture, and mix well by slowly stirring for 30 min.
19. Put the mixture into silicon capsules and polymerize the resin in an oven at 60°C for 2 days. The polymerized resin, rocket of resin, can be stored at 20°C–25°C for several years.

## Key resources table

**Table:** 

REAGENT or RESOURCE	SOURCE	IDENTIFIER
**Bacterial and virus strains**		

AAV2/1-SynTetOff-EGFP-APEX2	Furuta et al. (2022)	n/a

**Chemicals, peptides, and recombinant proteins**		

1 N sodium hydroxide (NaOH) solution	Nacalai Tesque	37421-05
2-Aminoethanol	Nacalai Tesque	23405-55
3,3′-Diaminobenzidine, tetrahydrochloride (DAB)	Dojindo	347-00904
10 N NaOH solution	Nacalai Tesque	94611-45
Agar	Nacalai Tesque	01028-85
Agarose	TaKaRa Bio	L03
Ammonium Nickel (II) Sulfate Hexahydrate (Nickel ammonium sulfate)	Nacalai Tesque	24217-82
Atipamezole (Antisedan®)	Nippon Zenyaku Kogyo	n/a
ß-D-glucose	Wako Pure Chemical Industries	049-31165
Biotin–NHS	Calbiochem	203112
Bovine serum albumin (BSA)	Nacalai Tesque	01863-77
Butorphanol tartrate (Vetorphale®)	Meiji Seika Pharma	n/a
Chloroform	Nacalai Tesque	08402-55
Dimethyl sulfoxide (DMSO)	Nacalai Tesque	13407-45
Di-sodium hydrogen phosphate 12-water (Na_2_HPO_4_·12H_2_O)	Nacalai Tesque	31722-45
D-Sorbitol	Nacalai Tesque	06286-55
Ethanol (EtOH) (99.5%)	Nacalai Tesque	14713-95
γ-cyclodextrin	Wako Pure Chemical Industries	037-10643
Gentamicin	Nichi-Iko Pharmaceutical	n/a
Glucose oxidase	Nacalai Tesque	16831-14
Glutaraldehyde (GA) (25% aqueous solution)	Nacalai Tesque	17003-92
Glycerol	Sigma-Aldrich	G9012
Hydrochloric acid (HCl)	Nacalai Tesque	18321-05
Hydrogen peroxide (H_2_O_2_) (31% aqueous solution)	Santoku	n/a
Lead nitrate	Nacalai Tesque	20231-02
Luveak-812	Nacalai Tesque	20829-05
Luveak-DDSA	Nacalai Tesque	14423-95
Luveak-DMP-30	Nacalai Tesque	14425-75
Luveak-MNA	Nacalai Tesque	14424-85
Medetomidine hydrochloride (Domitor®)	Nippon Zenyaku Kogyo	n/a
Midazolam (Dormicum®)	Astellas Pharma	n/a
Methyl-β-cyclodextrin	Tokyo Chemical Industry	M1356
Molecular Sieves 3A, mixed indicator	Nacalai Tesque	23356-05
Osmium (Ⅷ) Oxide (OsO_4_) (2% aqueous solution)	Nacalai Tesque	25746-06
Paraformaldehyde (PFA)	Merck Millipore	1.04005.1000
Phosphate buffered saline (10×) (pH 7.4) (10× PBS(–))	Nacalai Tesque	27575-31
Potassium chloride (KCl)	Nacalai Tesque	28514-75
Potassium dihydrogenphosphate (KH_2_PO_4_)	Nacalai Tesque	28721-55
Propylene oxide	Nacalai Tesque	29223-55
Siliconized L-25	Fuji Systems	0411002
Sodium azide (NaN_3_)	Nacalai Tesque	31233-55
Sodium dihydrogen phosphate dihydrate (NaH_2_PO_4_·2H_2_O)	Nacalai Tesque	31718-15
Sodium chloride (NaCl)	Nacalai Tesque	31320-05
Sodium pentobarbital (Somnopentyl®)	Kyoritsu Seiyaku	n/a
Sodium citrate	Nacalai Tesque	31404-15
Sucrose	Nacalai Tesque	30404-45
Tissue-Tek® O.C.T. Compound (OCT compound)	Sakura Finetek	4583
Tris(hydroxymethyl)aminomethane (Tris)	Nacalai Tesque	35434-21
Triton X-100	Nacalai Tesque	35501-15
Tyramine hydrochloride	Sigma-Aldrich	T2879-1G
Urea	Nacalai Tesque	35940-65
Uranyl acetate	Merck	Art. 8473

**Critical commercial assays**		

VECTASTAIN Elite ABC Kit	Vector	PK-6100

**Deposited data**		

CAD data of chamber frame for microscopic image acquisition of thick slices	Furuta et al. (2022)	Data S1
CAD data of stage adaptor for microscopic image acquisition of thick slices	Furuta et al. (2022)	Data S2

**Experimental models: Organisms/strains**		

Mouse: C57BL/6J (8–16 weeks old, male and female)	Nihon SLC	*C57BL/6JJmsSlc*

**Software and algorithms**		

ImageJ	Schneider et al. (2012)	https://imagej.nih.gov/ij/
Imaris	Bitplane	https://imaris.oxinst.jp/imaris-viewer
Leica Application Suite X	Leica Microsystems	https://www.leica-microsystems.com/products/microscope-software/p/leica-las-x-ls/

**Other**		

0.22 μm filter	Merck Millipore	SLGP033RB
13 mL centrifuge tube	SARSTEDT	60.541.545
16× multi-immersion objective lens	Leica Microsystems	HC FLUOTAR 16×/0.60 IMM CORR VISIR
25× water-immersion objective lens	Leica Microsystems	HC FLUOTAR L 25×/0.95 W VISIR
3D printing service	DMM.make	https://make.dmm.com
3D-printer	Keyence	AGILISTA-3200
40× objective lens	Olympus	UPlanApo ×40/0.85
63× oil-immersion objective lens	Leica Microsystems	HC PL APO 63×/1.40 Oil CS2
6-well culture plate	Greiner Bio-One	657160
Blu-Tack®	Bostik	n/a
Brightfield microscope	Olympus	BX-51
Carbon steel blades	Feather	FA-10B
Coverslip	Matsunami Glass	C025601
Cryomold	Sakura Finetek	4566
Diamond knife	Diatome	Ultra 45° 3.0 mm
Digital Microscope Color Camera	Olympus	DP74
Electro freeze	Yamato Koki	MC-802A
Glass capillary (diameter 2 mm)	Narishige	G-2
Glass slide	Matsunami Glass	S1225
Low temperature circulator bath	EYELA	CCA-1112A
Parafilm®	Bemis	PM-996
Picospritzer III	Parker Hannifin	n/a
Pressure-sensitive adhesive	CEMEDINE	NA-007
Rigid acrylic resin	Keyence	AR-M2
Sliding microtome	Leica Biosystems	SM2000R
Snow horn	Nippon Ekitan Corporation	n/a
Stereo microscope	Nikon	SMZ445
Superfrost APS-coated micro slide glass	Matsunami Glass	APS-01
Stereotaxic apparatus	Narishige	SR50
Synthetic diamond knife	Diatome	Histo 45° 4.0 mm
TCS SP8	Leica Microsystems	n/a
TEM grid	Nisshin EM	VECO GRID 150 mesh Cu
Transmission electron microscope	Hitachi	H-7650
Ultramicrotome	Leica Microsystems	Ultracut UCT
Vibratome	Dosaka EM	Linear PRO7N

## Materials and equipment

**Table:** Phosphate buffer (PB) (pH 7.4) 0.2 M

Reagent	Final concentration	Amount
NaH_2_PO_4_·2H_2_O	38 mM	11.8 g
Na_2_HPO_4_·12H_2_O	162 mM	116 g
ddH_2_O	n/a	up to 2 L
**Total**	**n/a**	**2 L**

The solution can be stored at 20°C–25°C for 6 months.

**Table:** Phosphate buffered saline (PBS) (pH 7.4) 10×

Reagent	Final concentration	Amount
NaCl	1.37 M	80 g
Na_2_HPO_4_·12H_2_O	81 mM	29 g
KCl	27 mM	2 g
KH_2_PO_4_	15 mM	2 g
ddH_2_O	n/a	up to 1 L
**Total**	**n/a**	**1 L**

The solution can be stored at 20°C–25°C for 6 months.

**Table:** Fixative solution

Reagent	Final concentration	Amount
PFA 16%	4% (w/v)	10 mL
GA 25%	0.2% (v/v)	320 μL
PB (pH 7.4) 0.2 M	0.1 M	20 mL
ddH_2_O	n/a	up to 40 mL
**Total**	**n/a**	**40 mL**

Filter the solution through filter paper.

Use the fixative solution within the day.

The final concentration of GA can be changed from 0.02% to 2% depending on experiments.

**CRITICAL:** PFA is toxic and teratogenic. Avoid inhalation or contact with skin, eyes and mucous membrane. Handle it inside a fume hood with appropriate protective gear.

**CRITICAL:** GA is a toxic and strong irritant, and should be handled inside a fume hood with appropriate protective gear. Contact with skin, eyes and mucous membrane must be avoided.

**Table:** Permeabilization buffer

Reagent	Final concentration	Amount
BSA	2% (w/v)	1 g
Triton X-100	0.2% (v/v)	100 μL
PBS (pH 7.4) 10×	1×	5 mL
ddH_2_O	n/a	up to 50 mL
**Total**	**n/a**	**50 mL**

Sterilize the solution with a 0.22 μm filter.

The solution can be stored at 4°C for 1 month.

**Table:** 2% BSA in 0.1 M PB

Reagent	Final concentration	Amount
BSA	2% (w/v)	1 g
PB (pH 7.4) 0.2 M	0.1 M	25 mL
ddH_2_O	n/a	up to 50 mL
**Total**	**n/a**	**50 mL**

Sterilize the solution with a 0.22 μm filter.

The solution can be stored at 4°C for 1 month.

**Table:** Glucose oxidase (GO) solution

Reagent	Final concentration	Amount
Glucose oxidase	1 mg/mL	1 mg
PB (pH 7.4) 0.2 M	0.1 M	500 μL
ddH_2_O	n/a	500 μL
**Total**	**n/a**	**1 mL**

Dispense to 100 μL each.

The solution can be stored at −80°C for 1 year.

**Table:** Biotin-NHS solution

Reagent	Final concentration	Amount
Biotin-NHS	95.9 mg/mL	3.5 mg
DMSO	n/a	36.5 μL
**Total**	**n/a**	**36.5 μL**

The solution should be prepared before use.

**Table:** Tyramine hydrochloride solution

Reagent	Final concentration	Amount
Tyramine hydrochloride	50 mg/mL	15 mg
DMSO	n/a	300 μL
**Total**	**n/a**	**300 μL**

The solution should be prepared before use.

**Table:** Biotinylated tyramine (BT) solution

Reagent	Final concentration	Amount
Biotin-NHS solution	n/a	36.5 μL
Tyramine hydrochloride solution	n/a	36.5 μL
2-Aminoethanol	n/a	7.3 μL
**Total**	**n/a**	**80.3 μL**

For a detailed procedure of preparation of BT solution, refer to Okamoto et al. (2021).

Mix well biotin–NHS solution and tyramine hydrochloride solution and incubate the mixture for 12–24 h at 20°C–25°C with rotation and protection from light.

Add 7.3 μL of monoethanolamine to the mixture, and incubate it for 4 h at 20°C–25°C with rotation and protection from light.

Complete reaction between biotin-NHS and tyramine hydrochloride yields 128 mM of BT in the solution.

The solution can be stored at −80°C for 3 years.

**Table:** Biotinylated tyramine-glucose oxidase (BT-GO) reaction mixture

Reagent	Final concentration	Amount
BT solution	1:5,000	1 μL
GO solution	3 μg/mL	15 μL
2% BSA in 0.1 M PB	n/a	5 mL
**Total**	**n/a**	**5 mL**

The solution should be prepared before use.

**CRITICAL:** The optimal concentration of BT solution should be determined for each experiment. We usually dilute the solution from 1:5,000 to 1:50,000 for the total.

**Table:** ß-D-glucose solution

Reagent	Final concentration	Amount
ß-D-glucose	200 mg/mL	200 mg
ddH_2_O	n/a	1 mL
**Total**	**n/a**	**1 mL**

Dispense to 100 μL each.

The solution can be stored at −80°C for 1 year.

**Table:** 4% PFA in 0.1 M PB

Reagent	Final concentration	Amount
PFA 16%	4% (w/v)	10 mL
PB (pH 7.4) 0.2 M	0.1 M	20 mL
ddH_2_O	n/a	10 mL
**Total**	**n/a**	**40 mL**

The solution can be stored at 4°C for 1 week.

**CRITICAL:** PFA is toxic and teratogenic. Avoid inhalation or contact with skin, eyes and mucous membrane. Handle it inside a fume hood with appropriate protective gear.

**Table:** Methyl-β-cyclodextrin 100 mM

Reagent	Final concentration	Amount
Methyl-β-cyclodextrin	100 mM	1.303 g
ddH_2_O	n/a	10 mL
**Total**	**n/a**	**10 mL**

Dispense to 1 mL each.

The solution can be stored at −20°C for 3 months.

**Table:** γ-cyclodextrin 100 mM

Reagent	Final concentration	Amount
γ-cyclodextrin	100 mM	1.297 g
ddH_2_O	n/a	10 mL
**Total**	**n/a**	**10 mL**

Dispense to 1 mL each.

The solution can be stored at −20°C for 3 months.

**Table:** Triton X-100 10%

Reagent	Final concentration	Amount
Triton X-100	10% (w/v)	5 g
ddH_2_O	n/a	up to 50 mL
**Total**	**n/a**	**50 mL**

The solution can be stored at 4°C for 3 months.

**Table:** Sca*l*eS0 solution

Reagent	Final concentration	Amount
D-sorbitol	20% (w/v)	20 g
Glycerol	5% (w/v)	5 g
Methyl-β-cyclodextrin 100 mM	1 mM	1 mL
γ-cyclodextrin 100 mM	1 mM	1 mL
DMSO	3% (v/v)	3 mL
10× PBS(–)	1×	10 mL
ddH_2_O	n/a	up to 100 mL
**Total**	**n/a**	**100 mL**

For a detailed procedure for preparation of this solution, refer to Miyawaki et al. (2016).

The solution can be stored at 4°C for 1 month.

***Note:*** The original Sca*l*eS0 solution contains N-acetyl-L-hydroxyproline (Skin Essential Actives, Hualian, Republic of China) (Hama et al., 2015). If this amino acid derivative is difficult to obtain from the manufacturer, N-acetyl-L-hydroxyproline should be omitted from the solution. N-acetyl-L-hydroxyproline provided by other manufactures can negatively affect tissue clarification (Miyawaki et al., 2016).

**Table:** Sca*l*eS4 solution

Reagent	Final concentration	Amount
Urea	4 M	24.02 g
D-sorbitol	40% (w/v)	40 g
Glycerol	10% (w/v)	10 g
Triton X-100 10%	0.2% (w/v)	2 mL
DMSO	25% (v/v)	25 mL
ddH_2_O	n/a	up to 100 mL
**Total**	**n/a**	**100 mL**

For a detailed procedure for preparation of this solution, refer to Miyawaki et al. (2016).

The solution can be stored at 4°C for 1 month.

**Table:** Sca*l*eS4 D25(0) solution

Reagent	Final concentration	Amount
Urea	4 M	24.02 g
D-sorbitol	40% (w/v)	40 g
Glycerol	10% (w/v)	10 g
DMSO	25% (v/v)	25 mL
ddH_2_O	n/a	up to 100 mL
**Total**	**n/a**	**100 mL**

For a detailed procedure for preparation of this solution, refer to Miyawaki et al. (2016).

The solution can be stored at 4°C for 1 month.

**Table:** Sca*l*eS4 gel

Reagent	Final concentration	Amount
Agarose	1.5% (w/v)	1.5 g
Sca*l*eS4D25(0)	100 mL	100 mL
**Total**	**n/a**	**100 mL**

For a detailed procedure for preparation of this solution, refer to Miyawaki et al. (2016).

The solution can be stored at 4°C for 1 month. Sca*l*eS4 gel is solidified at 4°C.

Remelt the gel by heating in a microwave before use.

**Table:** 30% Sucrose in 0.1 M PB

Reagent	Final concentration	Amount
Sucrose	30% (w/v)	30 g
0.2 M PB (pH 7.4)	0.1 M	50 mL
ddH_2_O	n/a	up to 100 mL
**Total**	**n/a**	**100 mL**

The solution can be stored at 4°C for 1 month.

**Table:** 75% Glycerol in PBS

Reagent	Final concentration	Amount
Glycerol	75% (v/v)	7.5 mL
PBS	25% (v/v)	2.5 mL
**Total**	**n/a**	**10 mL**

The solution can be stored at 20°C–25°C for 1 month.

**Table:** 2% BSA in PBS

Reagent	Final concentration	Amount
BSA	2% (w/v)	1 g
PBS (pH 7.4) 10×	1×	5 mL
ddH_2_O	n/a	up to 50 mL
**Total**	**n/a**	**50 mL**

Sterilize the solution with a 0.22 μm filter.

The solution can be stored at 4°C for 1 month.

**Table:** ABC solution

Reagent	Final concentration	Amount
Reagent A	1:50	20 μL
Reagent B	1:50	20 μL
2% BSA in PBS	n/a	1 mL
**Total**	**n/a**	**1 mL**

The solution should be prepared before use.

**CRITICAL:** ABC solution should be prepared before incubation with brain sections. Add 20 μL of Reagent A to 1 mL of 2% BSA in PBS, and then add 20 μL of Reagent B to the same solution, and mix immediately. Incubate the mixture for 30 min at 20°C–25°C with gentle agitation, and chill on ice.

**Table:** Tris-HCl (pH 7.6) 500 mM

Reagent	Final concentration	Amount
Tris	500 mM	60.57 g
ddH_2_O	n/a	up to 1 L
**Total**	**n/a**	**1 L**

Adjust pH to 7.6 with HCl.

The solution can be stored at 20°C–25°C for 1 year.

**Table:** Nickel ammonium sulfate 200 mM

Reagent	Final concentration	Amount
Nickel ammonium sulfate	200 mM	395 mg
ddH_2_O	n/a	5 mL
**Total**	**n/a**	**5 mL**

Dispense to 250 μL each.

The solution can be stored at −20°C for several years.

**CRITICAL:** Nickel ammonium sulfate is toxic and carcinogenic. Avoid inhalation or contact with skin, eyes and mucous membrane. Handle it inside a fume hood with appropriate protective gear.

**Table:** DAB-Ni^2+^ solution

Reagent	Final concentration	Amount
DAB	0.05% (w/v)	10 mg
Nickel ammonium sulfate 200 mM	2.5 mM	250 μL
Tris-HCl (pH 7.6) 500 mM	50 mM	2 mL
ddH_2_O	n/a	up to 20 mL
**Total**	**n/a**	**20 mL**

The solution should be prepared before use.

**CRITICAL:** DAB is carcinogenic. Waste should be oxidized before being discarded. Avoid inhalation or contact with skin, eyes and mucous membrane. Handle it inside a fume hood with appropriate protective gear.

**CRITICAL:** Nickel ammonium sulfate is toxic and carcinogenic. Avoid inhalation or contact with skin, eyes and mucous membrane. Handle it inside a fume hood with appropriate protective gear.

**Table:** 1% PFA in 0.1 M PB

Reagent	Final concentration	Amount
PFA 16%	1% (w/v)	2.5 mL
PB (pH 7.4) 0.2 M	0.1 M	20 mL
ddH_2_O	n/a	up to 40 mL
**Total**	**n/a**	**40 mL**

The solution can be stored at 4°C for 1 month.

**CRITICAL:** PFA is toxic and teratogenic. Avoid inhalation or contact with skin, eyes and mucous membrane. Handle it inside a fume hood with appropriate protective gear.

**Table:** 1% OsO_4_ in 0.1 M PB

Reagent	Final concentration	Amount
OsO_4_ 2%	1% (w/v)	5 mL
PB (pH 7.4) 0.2 M	0.1 M	5 mL
**Total**	**n/a**	**10 mL**

The solution should be prepared before use.

**CRITICAL:** OsO_4_ is highly volatile and reactive. Avoid inhalation or contact with skin, eyes and mucous membrane. Handle it inside a fume hood with appropriate protective gear.

**Table:** 2% uranyl acetate (UA) in 50% EtOH

Reagent	Final concentration	Amount
Uranyl acetate	2% (w/v)	20 mg
EtOH (99.5%)	50% (v/v)	0.5 mL
DDW	n/a	0.5 mL
**Total**	**n/a**	**1 mL**

The solution should be prepared before use.

**CRITICAL:** Uranyl acetate is a radioactive material and all necessary precautions should be taken when disposing. Handle it inside a fume hood with appropriate protective gear.

**Table:** 1% UA solution

Reagent	Final concentration	Amount
Uranyl acetate	1% (w/v)	10 mg
DDW	n/a	1 mL
**Total**	**n/a**	**1 mL**

The solution can be stored at 4°C in dark for 4 months.

**CRITICAL:** Uranyl acetate is a radioactive material and all necessary precautions should be taken when disposing. Handle it inside a fume hood with appropriate protective gear.

**Table:** Lead citrate solution

Reagent	Final concentration	Amount
Lead nitrate	2.66% (w/v)	1.33 g
Sodium citrate	3.52% (w/v)	1.76 g
1 N NaOH solution	0.16 M	8 mL
DDW	n/a	up to 50 mL
**Total**	**n/a**	**50 mL**

Dissolve lead nitrate and sodium citrate in DDW and leave it for 30 min before adding NaOH.

The solution can be stored at 4°C in dark for 4 months.

**CRITICAL:** To avoid production of lead carbonate, minimize contact of lead citrate solution with air.

**CRITICAL:** NaOH is corrosive. Handle it with appropriate protective gear. Contact with skin, eyes and mucous membrane must be avoided.

**Table:** 100% EtOH

Reagent	Final concentration	Amount
EtOH (99.5%)	n/a	100 mL
Molecular Sieves 3A	n/a	5 g
**Total**	**n/a**	**100 mL**

Mix thoroughly and allow the solution to stand for 24 h.

**CRITICAL:** Molecular sieves should be replaced when the beads color changes to beige demonstrating that the molecular sieves have reached equilibrium capacity.

**Table:** Epon 812 mixture

Reagent	Final concentration	Amount
Luveak-812	45.7% (v/v)	3.8 mL
Luveak-DDSA	24.0% (v/v)	2 mL
Luveak-MNA	28.8% (v/v)	2.4 mL
Luveak-DMP-30	1.4% (v/v)	0.12 mL
**Total**	**n/a**	**8.32 mL**

Mix well Luveak-812, Luveak-DDSA and Luveak-MNA by slowly stirring for 5 min.

Add Luveak-DMP-30 to the mixture and mix well for 30 min.

The mixture can be stored at −80°C for 6 months.

**Table:** Epoxy resin/propylene oxide mixture

Reagent	Final concentration	Amount
Epon 812 mixture	50% (v/v)	5 mL
Propylene oxide	50% (v/v)	5 mL
**Total**	**n/a**	**10 mL**

Mix well by slowly stirring.

The solution should be prepared before use.

## Step-by-step method details

### Tissue slice preparation

**Timing: 2 days**

This step describes how to prepare mouse brain slices for the successive LM/EM imaging with Sca*l*eSF tissue clearing.

1. Sacrifice mice injected with the AAV2/1-SynTetOff-EGFP-APEX2 vector by ip injection of overdose of Somnopentyl® (200 mg/kg).

**CRITICAL:** Perform steps 2 through 3 in a fume hood to limit the exposure to PFA and GA.

2. Perfusion fixation.
  a. Open the thoracic cavity with surgical scissors.
  b. Make an incision to the right atrial appendage with surgical scissors.
  c. Insert a 22-gauge needle into the left ventricle of heart.
  d. Perfuse with 20 mL of ice-cold PBS using a 20 mL syringe.
  e. Perfuse with 20 mL of the ice-cold fixative solution with another 20 mL syringe.
3. Excise brains using surgical scissors and tweezers.

**CRITICAL:** From steps 4 to 5, samples should be protected from light.

4. Immerse the brain in the fixative solution and rock gently for 16 h–20 h at 4°C (50 rpm–100 rpm).

**CRITICAL:** Samples should not be stored for extended periods of time. Procced to the next step immediately.

5. Brain slice preparation.
  a. Embed the brain tissues in 4% agar in PBS in a standard 6-well culture plate.
    ***Note:*** The temperature of the agar solution should be between 40°C and 45°C.
    ***Note:*** The viscous property by agaropectin, a major component of agar, improves ease of cutting.
  b. Allow the agar to solidify on ice for 30 min.
  c. Fill the vibratome bath with crashed ice.
  d. Stick a piece of mending tape on the bottom of the specimen tray.
  e. Attach the tray to the bath.
  f. Trim the agar block with a razor blade to square off the sides.
  g. Mount the agar block onto the tape with superglue.
  h. Pour ice-cold 0.1 M PB to the specimen tray.
  i. Set the sectioning speed to 0.14 mm/s with 1.4 mm amplitude.
  j. Set the vibratome frequency to 75 Hz–77 Hz.
  k. Cut 1-mm-thick brain slices.
  l. Collect the slices in ice-cold PBS.
    **Pause point:** The slices can be stored in PBS for 16 h–20 h at 4°C.
    **CRITICAL:** NaN_3_ as a preservative should be avoided. NaN_3_ inactivates the enzymatic activity of APEX2.

### APEX2/BT-GO reaction

**Timing: 2 days**

**Figure 2:**
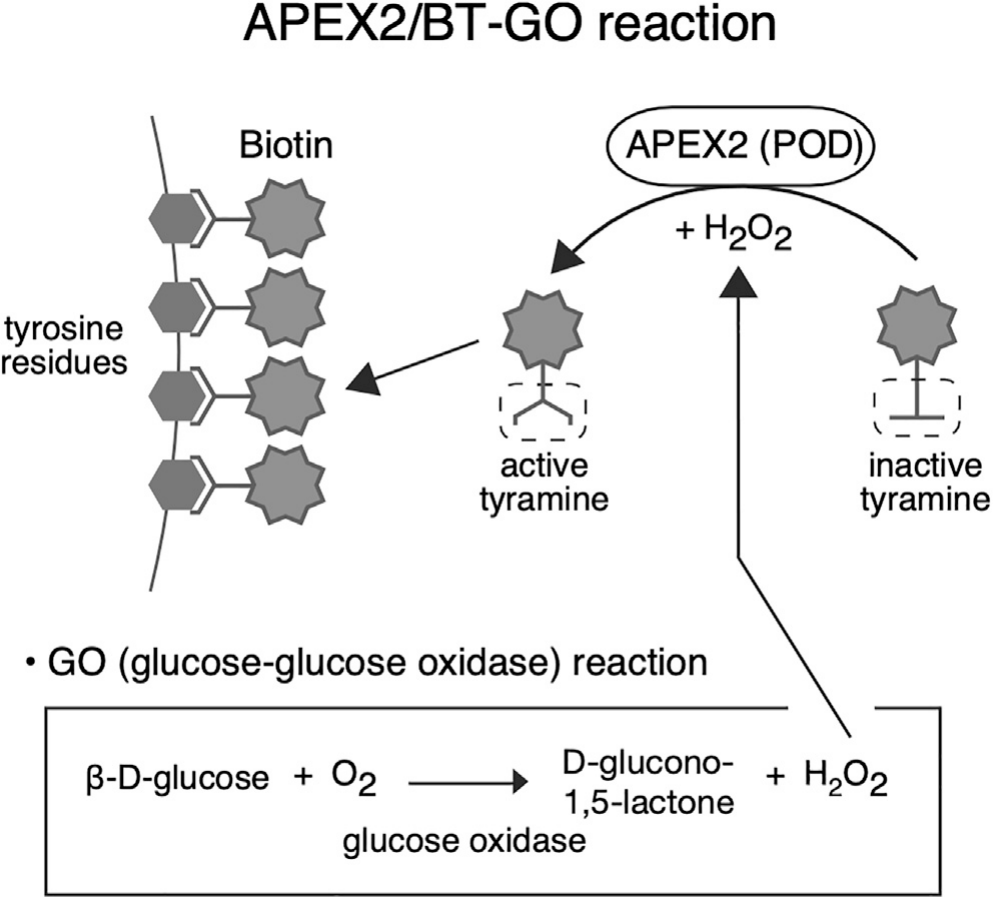
Schematic diagram of APEX2/BT-GO reaction APEX2/BT-GO is a TSA system that utilizes peroxidase activity of APEX2 and H_2_O_2_ produced during oxidation of glucose by glucose oxidase (GO reaction) to deposit biotinylated tyramine (BT) onto tissues. APEX2 reacts with the H_2_O_2_ and oxidizes the phenolic part of BT to produce highly reactive intermediates, which in turn covalently bind to electron-rich moieties such as tyrosine residues at or near the APEX2. POD: peroxidase.

In this step, we explain the procedure for APEX2/BT-GO reaction, where biotin molecules are deposited with tyramide signal amplification (TSA) reaction using the peroxidase activity of APEX2 (Figure 2) (Furuta et al., 2022). This step is essential for LM/EM dual labeling resistance for Sca*l*eSF tissue clearing. All incubations are performed in a standard 6-well culture plate at 20°C–25°C, except where noted.

**CRITICAL:** APEX2/BT-GO should be performed prior to tissue clearing with Sca*l*eSF. Peroxidase activity of APEX2 markedly declines following Sca*l*eSF treatment (Figure S1).

**CRITICAL:** Samples should be protected from light.

6. Permeabilization.
  a. Immerse the slices in 10 mL of permeabilization buffer in a 13 mL centrifuge tube.
  b. Rock gently for 4 h (50 rpm–100 rpm).

**CRITICAL:** Brain tissues should be shaken thoroughly.

7. Wash.
  a. Transfer the slices in 8 mL of 0.1 M PB.
  b. Wash the slices with 8 mL of 0.1 M PB for 5 min three times with gentle rocking (50 rpm–100 rpm).
8. BT penetration.
  a. Transfer the slices in 1200 μL of BT-GO reaction mixture in a 2 mL safe-lock microcentrifuge tube.
  b. Rock gently for 4 h (50 rpm–100 rpm).

**CRITICAL:** Brain tissues should be shaken thoroughly.

9. BT deposition.
  a. Add 12 μL of ß-D-glucose solution to the BT-GO reaction mixture.
  b. Rock gently for 2 h (50 rpm–100 rpm).

**CRITICAL:** Brain tissues should be shaken thoroughly.

10. Wash.
  a. Transfer the slices in 8 mL of PBS.
  b. Wash the slices with 8 mL of PBS for 15 min twice with gentle rocking (50 rpm–100 rpm).
11. Fixation.
  a. Immerse the slices in 8 mL of 4% PFA in 0.1 M PB.
  b. Rock gently for 16 h–20 h at 4°C (50 rpm–100 rpm).
12. Wash.
  a. Transfer the slices in 8 mL of PBS.
  b. Wash the slices with 8 mL of PBS for 5 min twice with gentle shaking (50 rpm–100 rpm).

**Pause point:** The slices can be stored in 0.02% NaN_3_ in PBS at 4°C for 1 week.

### Tissue clearing

**Timing: 10.5–14.5 h**

**Figure 3:**
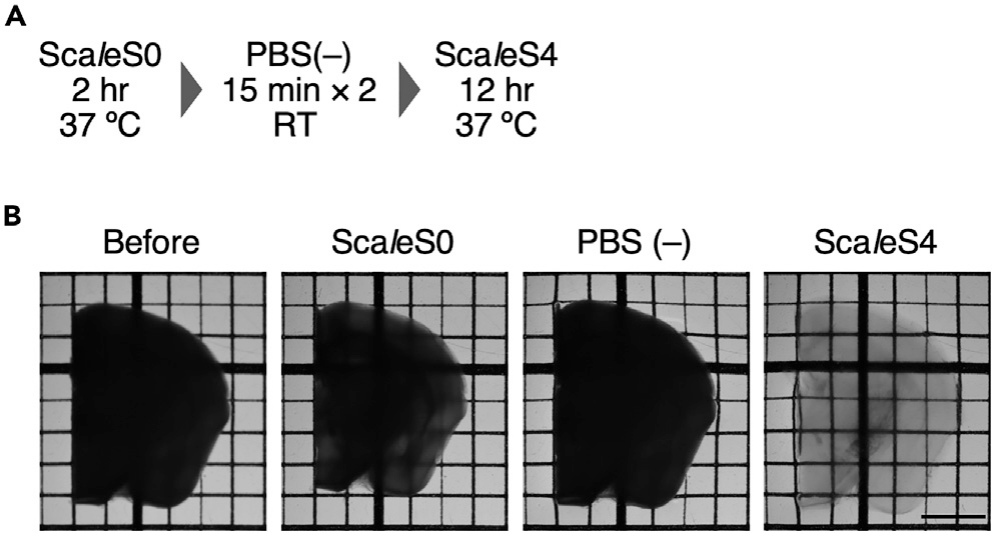
Sca*l*eSF tissue clearing in a mouse brain slice of 1-mm thickness (A) The schedule for Sca*l*eSF tissue clearing. (B) Transmission images of a mouse brain slice during Sca*l*eSF tissue clearing. Images are acquired before and after Sca*l*eS0 treatment, after washes with PBS(–), and after an incubation in Sca*l*eS4 solution. The grid interval is 1 mm. Scale bar, 2 mm.

This step describes the procedure for Sca*l*eSF tissue clearing. The clearing schedule is shown in Figure 3A. All reactions are performed in a standard 6-well culture plate.

**CRITICAL:** Samples should be protected from light.

13. Sca*l*eSF tissue clearing (Figure 3B).
  a. Warm Sca*l*eS0 and Sca*l*eS4 solutions to 37°C.
  b. Immerse the brain slices in 8 mL of Sca*l*eS0 solution, and rock gently for 2 h at 37°C (90 rpm).
  c. Transfer the slices in 8 mL of 1× PBS (–), and wash twice for 15 min with gentle rocking at 20°C–25°C (50 rpm–100 rpm).
  d. Transfer the slices in 8 mL of Sca*l*eS4 solution, and rock gently for 8 h–12 h at 37°C (90 rpm).

### Brain slices mounting

**Timing: ∼2 h**

This step describes how to mount the cleared brain slices on the imaging chamber.

14. Brain slice mounting.
  a. Place the cleared brain slices on the bottom coverslip of the imaging chamber.
  b. Remove excessive Sca*l*eS4 solution around the cleared slice.
15. Sca*l*eS4 gel embedding.
  a. Drop Sca*l*eS4 gel on the cleared slice to fill the imaging chamber.
    ***Note:*** The temperature of the Sca*l*eS4 gel should be kept at 37°C.
  b. Mount a coverslip on the imaging chamber, and place a piece of Kimwipe paper and a slide glass on the coverslip in this order.
  c. Transfer the imaging chamber in a refrigerator at 4°C.
  d. Place metal weights on the slide glass, and allow the gel to solidify for 30 min.
  e. Remove the metal weights, slide glass, Kimwipe paper and coverslip from the imaging chamber, and wipe away excess Sca*l*eS4 gel.
16. Tissue equilibration.
  a. Attach the imaging chamber to the bottom of a 60-mm glass petri dish with Blu-Tack®. Adhesion at multiple points is required.
  b. Pour Sca*l*eS4 solution to the dish, and incubate the embedded slice in the solution for 60 min at 20°C–25°C with gentle agitation (40 rpm–60 rpm).
  c. Substitute with fresh Sca*l*eS4 solution.

**CRITICAL:** Air bubbles on the sample surface should be removed.

***Optional:*** Using microscope stage adaptors, the imaging chambers can be mount on a microscope stage directly. In this case, the coverslip should remain attached on the imaging chamber.

### Confocal laser scanning microscopy (CLSM) imaging in cleared tissues

**Timing: 2–24 h**

17. Set the correction collar of a 16× multi-immersion objective lens (numerical aperture (NA) = 0.60, working distance [WD] = 2.5 mm) to 1.47.

**CRITICAL:** Refractive index (RI) mismatch-induced aberrations can highly degrade the image formation. Sca*l*eS4 solution has a RI of around 1.47 (Hama et al., 2015; Miyawaki et al., 2016).

18. Mount the imaging chamber on a microscope stage.
19. Immerse the objective lens in Sca*l*eS4 solution, and make it approach to the cleared slice slowly.

**CRITICAL:** Air bubbles trapped on the tip of the objective lens should be removed.

20. Adjust imaging acquisition settings such as laser power, scan speed, pinhole diameter, detector gain, amplifier offset/gain, *xy* and *z*-axis resolution and bit intensity resolution.
21. Collect images by a CLSM, and record captured images.
22. Image processing is done with Leica Application Suite X and ImageJ.

### Re-sectioning

**Timing: 2 days**

Here, we explain the procedure for re-sectioning from the slices imaged with CLSM. All reactions are performed in a standard 6-well culture plate at 20°C–25°C, except where noted.

23. DeSca*l*e.
  a. Remove Sca*l*eS4 gel around the imaged brain slices.
  b. Immerse the slice in 8 mL of 1× PBS (–), and wash twice for 15 min with gentle rocking at 20°C–25°C (40 rpm–60 rpm).
24. Fixation.
  a. Immerse the slices in 8 mL of 4% PFA in 0.1 M PB, and rock gently for 16 h–20 h at 4°C (50 rpm–100 rpm).
25. Wash.
  a. Transfer the slices in 8 mL of 1× PBS (–), and wash the slices with 8 mL of 1× PBS (–) for 5 min twice with gentle shaking (40 rpm–60 rpm).

**Pause point:** The slices can be stored in 0.02% NaN_3_ in PBS at 4°C for 1 week.

26. Cryoprotection.
  a. Immerse the slices in 8 mL of 30% sucrose in 0.1 M PB at 4°C until sunk (a sign of complete immersion).

**CRITICAL:** Permeate brain slices with the cryoprotectant agent adequately. Adequate cryoprotection is of importance to reduce ice crystal formation and preserve cellular structure.

**Pause point:** The slices can be stored in 30% sucrose in 0.1 M PB for 16 h–20 h at 4°C.

27. Freezing brain slices.
  a. Immerse the slices in OCT compound for 15 min.
  b. Place the slice in a cryomold containing OCT compound.
  c. Cool isopentane in a metal container on liquid nitrogen.
    **CRITICAL:** Cool isopentane almost to its freezing temperature. Rapid freezing is of importance to reduce ice crystal formation and preserve cellular structure.
  d. Immerse the cryomold in the cooled isopentane to freeze.
  e. Transfer the cryomold in a freezer at −20°C.
    **Pause point:** The blocks can be stored at −20°C for a week.

28. Cryosectioning.
  a. Mount the block onto the stage of a freezing microtome with OCT compound.
  b. Quickly freeze the OCT compound using dry ice powder and leave the block in place for 5 min.
  c. Cut the slice into 40-μm-thick sections and collect in ice-cold 0.1 M PB.

**Pause point:** The sections can be stored in 0.02% NaN_3_ in PBS at 4°C for 1 week.

***Optional:*** Imaged slices can be embedded in agar or gelatin solution, and re-sectioned with a vibratome.

### CLSM imaging in re-sections

**Timing: 1 day**

Here, we explain the procedure for CLSM imaging in re-sections with a high NA objective lens, which allows for high-resolution imaging of targeted structures.

**CRITICAL:** Be sure to keep re-sections wet during this step.

29. Section mounting.
  a. Place a re-section on a slide with a brush.
  b. Apply 75% glycerol in PBS to the re-section.
  c. Lower a coverslip onto 75% glycerol in PBS to avoid trapping any air bubbles.
30. CLSM imaging.
  a. Mount the slide on a microscope stage.
  b. Adjust imaging acquisition settings such as laser power, scan speed, pinhole diameter, detector gain, amplifier offset/gain, *xy* and *z*-axis resolution and bit intensity resolution.
  d. Collect images by a CLSM equipped with high NA objective lenses, such as a 25×/NA 0.95 water-immersion and 63×/NA 1.40 oil-immersion objective lenses.
  e. Record captured images.
  f. Image processing is done with Leica Application Suite X and ImageJ.
31. Wash.
  a. Remove the coverslip from the slide.
  b. Immerse the re-section in 8 mL of PBS in a standard 6-well culture plate, and wash twice for 15 min with gentle rocking at 20°C–25°C (40 rpm–60 rpm).

**Pause point:** The sections can be stored in 0.02% NaN_3_ in PBS at 4°C for 1 week.

### ABC/DAB-Ni^2+^ reaction

**Timing: 2 days**

This step describes the procedure for ABC/DAB-Ni^2+^ reaction. All incubations are performed in a standard 12-well culture plate at 4°C, except where noted.

32. Wash.
  a. Immerse the sections in 3 mL of PBS, and wash twice for 10 min with gentle rocking (50 rpm–100 rpm).
33. ABC reaction.
  a. Immerse the sections in 500 μL of ABC solution in a standard 24-well plate, and rock gently for 24 h (50 rpm–100 rpm).
34. Wash.
  a. Immerse the sections in 3 mL of PBS, and wash twice for 10 min with gentle rocking (50 rpm–100 rpm).
35. DAB-Ni^2+^ reaction.
  a. Immerse the sections in 1 mL of DAB-Ni^2+^ solution in a standard 24-well plate on ice, and incubate for 10 min on ice with gentle agitation (40 rpm–60 rpm).
  b. Add 3 μL of 0.1% H_2_O_2_ into DAB-Ni^2+^ solution, and incubate on ice with gentle agitation until color development (40 rpm–60 rpm).
  c. Immerse the sections in 3 mL of 2% NaN_3_ in PBS, and incubate for 15 min at 20°C–25°C with gentle agitation (40 rpm–60 rpm).
36. Wash.
  a. Immerse the sections in 3 mL of PBS at 4°C and wash twice for 10 min with gentle rocking (50 rpm–100 rpm).

**Pause point:** The sections can be stored in 1% PFA in 0.1 M PB at 4°C for 1 month.

### Processing sections for EM

**Timing: 3 days**

This step describes processing tissue sections for ultra-thin sectioning and EM observation. This includes osmification, uranyl acetate staining and epoxy-resin embedding. All incubations are performed at 20°C–25°C, except where noted.

37. Wash.
  a. Immerse the sections in 3 mL of 0.1 M PB, and wash twice for 10 min.
38. Osmification.
  a. Immerse the sections in 1 mL of 1% OsO_4_ in 0.1 M PB, and incubate for 2 h in dark.

**CRITICAL:** The reaction should be protected from light.

39. Wash.
  a. Immerse the sections in 3 mL of 0.1 M PB, and wash three times for 10 min.
  b. Immerse the sections in 10 mL of ddH_2_O, and wash five times for 2 min with gentle rocking (50 rpm–100 rpm).
40. Block staining.
  a. Immerse the sections in 1 mL of 2% UA in 50% EtOH, and incubate for 30 min in dark.

**CRITICAL:** The reaction should be protected from light.

41. Dehydration.
  a. Incubate the sections in 5 mL of increasing concentrations of ethanol in ddH_2_O for 20 min in the following order: 50%, 70%, 90%, 99.5%, 99.5%, 100%, and 100%. Incubations are performed in a glass vial.
  b. Incubate the sections in 10 mL of propylene oxide for 20 min twice.
42. Epoxy resin embedding.
  a. Immerse the sections in 5 mL of Epoxy resin/propylene oxide mixture and incubate for 1 h.
  b. Immerse the sections in Epoxy resin and incubate for 16 h–20 h.
  c. Place the sections onto silicone-coated slide glass, and mount a silicone-coated coverslip on the slide.
  d. Place metal weights on the coverslip to make the sections flat.
  e. Remove the resin oozing from the slide.
  f. Polymerize the resin by heating in an oven at 60°C for 2 days.

**Pause point:** The flat-embedded sections can be stored at 20°C–25°C in a desiccator for several months.

### Ultra-thin sectioning

**Timing: 1 day**

Here, we describe how to prepare ultra-thin sections.

43. Remove the coverslip from the slide with a razor blade. The embedded sections stick to either the coverslip or the slide.
44. Identify the region of interest (ROI) with a brightfield microscope.
45. Trim the ROI using a razor blade under a stereo microscope.
46. Mount the trimmed resin block on a flat-tipped rocket of the resin with a glue.
47. Trim away excess resin with a razor blade.
48. Mount the rocket on the stage of an ultramicrotome, and align the block face with a synthetic diamond knife.
49. Remove the empty resin from the top of the sample using the synthetic diamond knife to expose the surface of the tissue.
50. Make ultrathin sections (60–80-nm thickness) with a diamond knife.
51. Collect short ribbons of ultrathin sections on TEM grids of copper, and let them dry.
52. Drop 1% UA solution on Parafilm®, and place the grids on the droplet for 20 min.
53. Wash the grids three times by dipping them into 10 mL of ddH_2_O.
54. Place the grids on the droplet of lead citrate solution on Parafilm® for 2 min.
55. Wash the grids three times by dipping into 10 mL of ddH_2_O, and let them dry.

**Pause point:** The ultrathin sections can be stored in a grid box at 20°C–25°C for several years.

### Transmission electron microscopy (TEM) imaging

**Timing: 1 day**

This step describes a procedure for TEM imaging. We usually acquire images at a resolution of 1.2–16.1 nm/pixel.

56. Set an accelerating voltage of 80 kV and a filament voltage of approximately 20 V.
57. Load the grids onto the grid holder.
58. Locate the ROI under a lower magnification mode.
59. Increase the magnification, and acquire individual images.
60. Record captured images.
61. Image processing is done using ImageJ software.

## Expected outcomes

**Figure 4:**
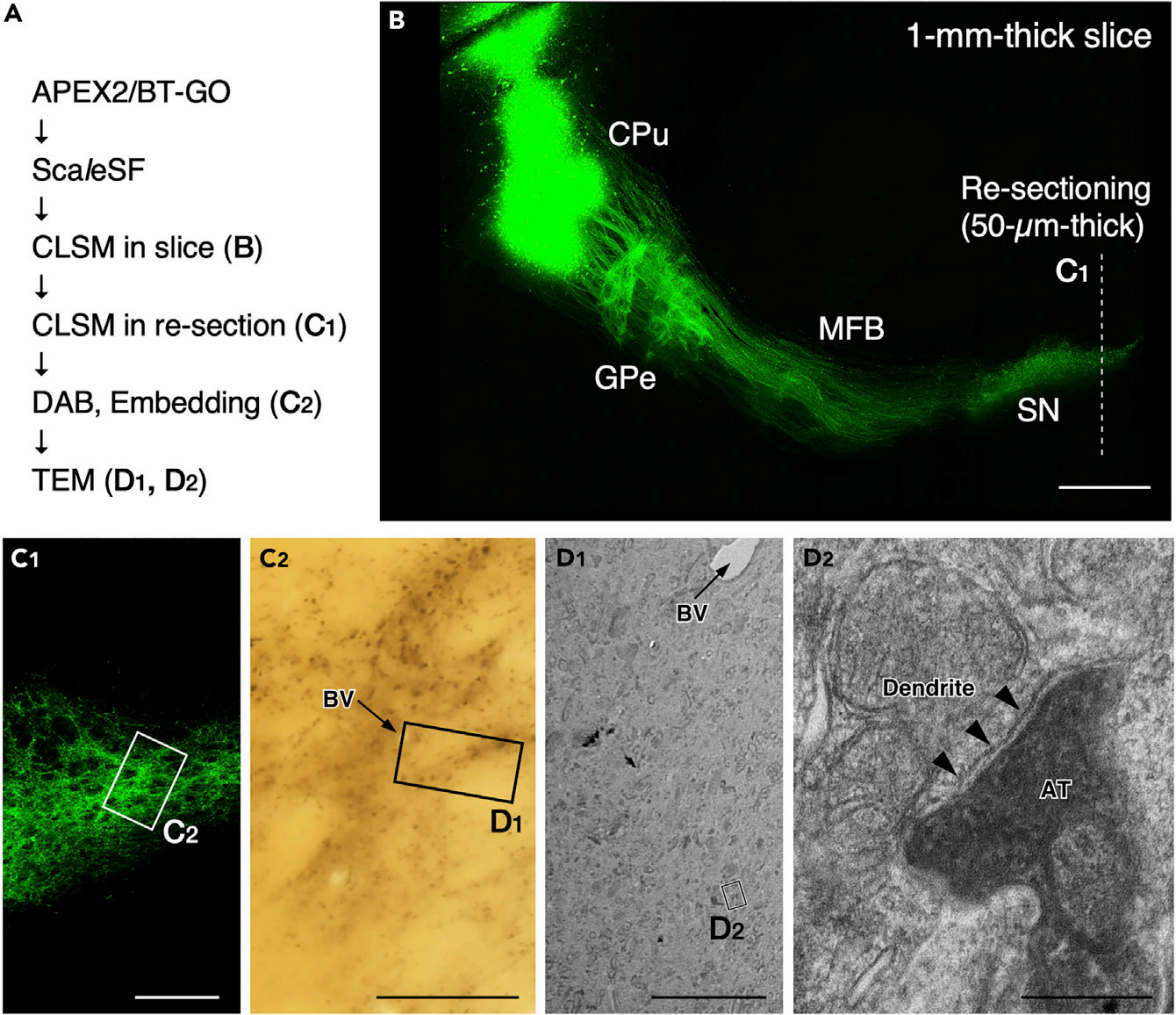
Multi-scale LM/EM neuronal imaging of the mouse striatofugal projection system (A) The procedure for multi-scale LM/EM neuronal imaging. (B) Neural circuit mapping in a 1-mm-thick brain slice cleared with Sca*l*eSF. The AAV2/1-SynTetOff-EGFP-APEX2 vector is injected into the CPu. A re-section of 50-μm thickness is cut along the dotted line. Scale bar, 500 μm. MFB: medial forebrain bundle. (C_1_) High-resolution subcellular imaging in the re-section at the level of SN. Scale bar, 200 μm. (C_2_) DAB-Ni^2+^ labeling with APEX2/BT-GO reaction. The re-section is embedded in epoxy resin. Scale bar, 50 μm. BV: blood vessel. (D_1_) A TEM image of the rectangle in (C_2_). Scale bar, 10 μm. Arrows in (C_2_ and D_1_) indicate the identical blood vessel. (D_2_) A high magnification image of the rectangle in (D_1_). Arrowheads indicate postsynaptic membrane. Scale bar, 500 nm. AT: axon terminal. Reprinted and modified from Furuta et al. (2022) under the Creative Commons Attribution 4.0 International License (CC BY 4.0; https://creativecommons.org/licenses/by/4.0/).

By following the clearing protocol of Sca*l*eSF, one can make mouse brain slices of 1-mm thickness transparent within 14.5 h after transient shrinkage and expansion (Figure 3). A multi-scale neuronal imaging in the mouse striatofugal projection system is shown in Figure 4. The mouse striatofugal projection is labeled by an injection of a LM/EM dual labeling vector, AAV2/1 SynTetOff EGFP-APEX2, into the caudate-putamen (CPu). Following tissue slice preparation, APEX2/BT-GO reaction and Sca*l*eSF tissue clearing, the cleared brain slice is subjected to neural circuit mapping using a CLSM equipped with a multi-immersion objective lens of long WD (Figure 4A). EGFP-labeled axons arising from the CPu extended caudally to the brainstem, forming dense terminal fields in the external segment of the globus pallidus (GPe) and substantia nigra (SN). After re-sectioning the imaged brain slice perpendicularly, high-resolution image stacks collected with a high NA objective lens show varicose axon arborizations of the EGFP-labeled neurons in the SN (Figure 4C_1_). Then, the imaged re-section is processed for ABC/DAB-Ni^2+^ reaction using biotin molecules deposited by APEX2/BT-GO reaction. One should see DAB-Ni^2+^ precipitates in the re-section. Following epoxy resin embedding (Figure 4C_2_) and ultra-thin sectioning, an axon terminal filled with DAB-Ni^2+^ precipitates is imaged with TEM (Figures 4D_1_ and 4D_2_). TEM imaging shows a symmetric synapse, which is characterized by the absence of PSD and the narrow synaptic cleft, on a dendrite of a SN neuron (Figure 4D_2_). For accurately tracking targets across multiple spatial scales, we took advantage of GFP fluorescence, DAB-Ni^2+^ labeling and/or endogenous landmarks such as shape of brain structure and blood vessels. Unambiguous correlation of LM and EM datasets was achieved by LM/EM dual labeling with a correlative light and electron microscopy (CLEM) probe, EGFP-APEX2.

## Limitations

In the present protocol, we provide a detailed procedure for successive LM/EM for mouse neuronal cells with Sca*l*eSF tissue clearing. Sca*l*eSF is an isometric and rapid tissue clearing method and archives a high-level of preservation of ultrastructure and fluorescence signals as well as potent clearing capability. However, two limitations remain in Sca*l*eSF tissue clearing. The first is the ultrastructural preservation. A lipid-extracting detergent in Sca*l*eS4 solution and/or incubations of brain tissues at 37°C for a total of 10–14 h can potentially damage the ultrastructural integrity. Indeed, we found a slight but statistically significant degradation of the ultrastructure accompanied by Sca*l*eSF tissue clearing (Furuta et al., 2022). The second is the clearing capability of Sca*l*eSF: brain slices of 1-mm thickness, but not a whole brain, can be cleared with the clearing protocol of Sca*l*eSF. Although 1-mm-thick brain slices can provide good knowledge of dendritic and local axonal arbors, information about long-range projections is fragmentary and incomplete in these slices (Stepanyants et al., 2009).

In the protocol described here, LM/EM dual labeling is implemented by coupling a genetically encoded CLEM probe, EGFP-APEX2, with APEX2/BT-GO reaction. Despite its potent LM/EM dual labeling and resistance for Sca*l*eSF tissue clearing, APEX2/BT-GO reaction itself and/or permeabilization for intracellular staining with a lipid-extracting detergent can potentially negatively affect the cellular ultrastructure. The LM/EM dual labeling gave strong EM contrast that was introduced in the form of osmiophilic polymers throughout the cytoplasm (Figure 3). Although the cytoplasmic labeling facilitates the identification of targeted structures, the labeling may interfere with interrogation of ultrastructural features of synapses such as active zones, PSDs and synaptic vesicle morphologies.

## Troubleshooting

### Problem 1

Brain tissues look opaque after clearing (step 13).

### Potential solution

Use Sca*l*eS solutions that are freshly prepared. Sca*l*eS solutions can be stored up to 1 month at 4°C. Consider prolonged incubation in Sca*l*eS4 solution.

### Problem 2

Weak or very light fluorescent signal (step 21).

### Potential solution

Use Sca*l*eS solutions that are freshly prepared. Fluorescent proteins are sensitive to certain environmental conditions, such as pH, ions, and redox state.

### Problem 3

3D reconstruction of CLSM images is not acceptable (step 22).

### Potential solution

Set the correction collar of the multi-immersion objective lens to 1.47. Sca*l*eS4 solution has a RI of 1.47 (Hama et al., 2015; Miyawaki et al., 2016). RI mismatch-induced aberrations can highly disturb the image formation.

### Problem 4

Ice crystal damage is evident in re-sections (step 28).

### Potential solution

Cool isopentane almost to its freezing temperature. Permeate brain tissues with the cryoprotectant agent adequately. This problem is caused by too slow a freezing rate and/or inadequate cryoprotection of tissues.

### Problem 5

No DAB-Ni^2+^ precipitation (step 35).

### Potential solution

NaN_3_ as a preservative should be avoided before APEX2/BT-GO reaction. NaN_3_ inactivates the enzymatic activity of APEX2. Use BT-GO reaction mixture that is freshly prepared.

### Problem 6

Weak or very light DAB-Ni^2+^ reaction (step 35).

### Potential solution

Consider increasing the concentration of BT solution. We usually dilute the solution from 1:5,000 to 1:50,000 for the total. Try to increase the incubation period of DAB-Ni^2+^ solution. Additional H_2_O_2_ can be supplied to the DAB-Ni^2+^ solution during color development. Following longer storage before APEX2/BT-GO reaction, the staining usually becomes less intense.

### Problem 7

Strong DAB-Ni^2+^ reaction is observed only at the edge of tissues (step 35).

### Potential solution

This problem is caused by poor penetration of BT into brain tissues. Verify that brain tissues are shaken thoroughly in both permeabilization buffer and BT-GO reaction mixture. For penetration of BT, it is important to shake brain tissues thoroughly in both solutions. Consider increasing the incubation period of permeabilization buffer and/or BT-GO reaction mixture. Prolonged incubation in permeabilization buffer, however, can potentially damage ultrastructure in brain tissues.

### Problem 8

No DAB-Ni^2+^ staining in the center of the infected cells (step 35).

### Potential solution

Consider decreasing the AAV labeling density. This problem can occur when the labeling density of APEX2 is extremely high. This might be due to the local reactant depletion (Zhang et al., 2019).

### Problem 9

Very high background (step 35).

### Potential solution

Try to decrease the concentration of BT solution. The optimal concentration of BT solution should be determined for each experiment. We usually dilute the solution from 1:5,000 to 1:50,000 for the total.

### Problem 10

Strong DAB-Ni^2+^ reaction is observed in vasculature (step 35).

### Potential solution

Perfuse brain tissues with ice-cold PBS adequately to remove red blood cells. Endogenous peroxidases in red blood cells retained in vasculature can react with H_2_O_2_ to deposit BT onto tissues.

### Problem 11

Poor ultrastructural preservation (step 56).

### Potential solution

Consider increasing the concentration of GA in the fixative solution. High concentration of GA fixation provides superior ultrastructure preservation in brain slices cleared with Sca*l*eSF (Furuta et al., 2022). Try to decrease the incubation period of Sca*l*eS4 solution and/or permeabilization buffer. These solutions contain a lipid extracting detergent that deleteriously affects membrane integrity. Sample handling at low temperature can minimize ultrastructural damage.

## Resource availability

### Lead contact

Further information and requests for resources and reagents should be directed to and will be fulfilled by the lead contact, Hiroyuki Hioki (h-hioki@juntendo.ac.jp).

### Materials availability

This study did not generate new unique reagents.

## Data Availability

Original/source data for 3D CAD date in the paper is available in [https://doi.org/10.1016/j.isci.2021.103601].
