# Supplementary material for: Protocol for multi-scale light microscopy/electron microscopy neuronal imaging in mouse brain tissue
Source: STAR Protoc. 2022 Aug 18;3(3):101508. doi: 10.1016/j.xpro.2022.101508 (PMC9405099; doi:10.1016/j.xpro.2022.101508)
Supplement: Document S1. Figure S1 [file mmc1.pdf]

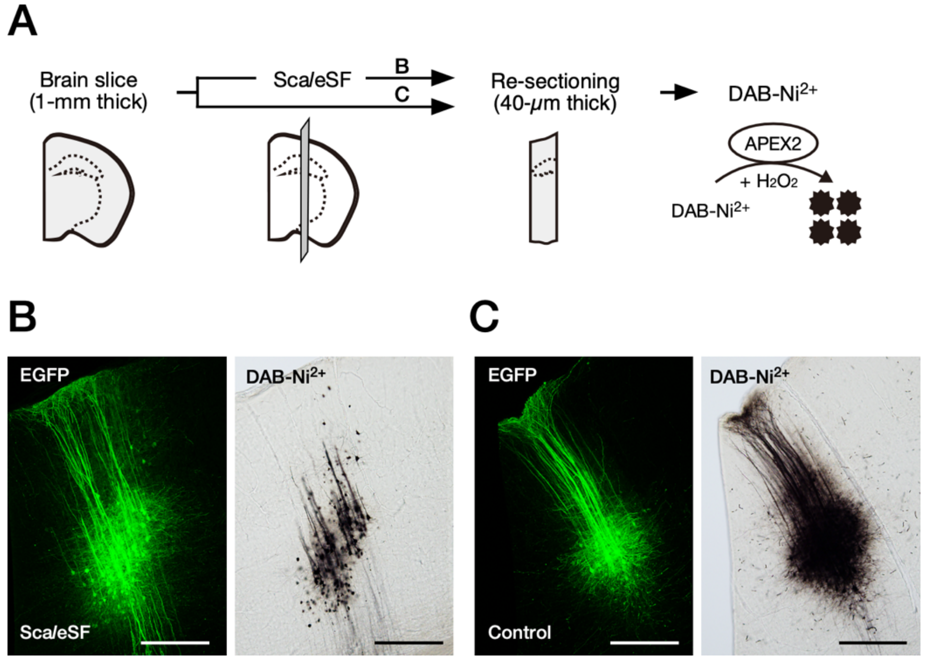

Figure S1. A declined peroxidase activity of APEX2 following Sca*l*eSF treatment, Related to Step 6 to 12.

(A) A schematic diagram of DAB-Ni^{2+} labeling with APEX2.

(B and **C)** DAB-Ni^{2+} labeling with APEX2 in mouse brain sections prepared from brain slices cleared with Sca*l*eSF (B) or stored in PBS(–) (C). Correlated fluorescence (left) and bright-field (right) images in neuronal cells labeled with EGFP-APEX2 fusion protein. Following CLSM imaging, re-sections are developed in DAB-Ni^{2+} solution. Scale bars, 500 µm.

Reprinted and modified from Furuta et al. (2022) under the Creative Commons Attribution 4.0 International License (CC BY 4.0; https://creativecommons.org/licenses/by/4.0/).
